# New Achievements from Molecular Biology and Treatment Options for Refractory/Relapsed Ovarian Cancer—A Systematic Review

**DOI:** 10.3390/cancers15225356

**Published:** 2023-11-10

**Authors:** Cornelia Bachmann

**Affiliations:** Department of Womens’ Health, University Tübingen, 72070 Tübingen, Germany; cornelia.bachmann@med.uni-tuebingen.de

**Keywords:** ovarian cancer, relapse, refractory ovarian cancer, immunotherapy, biomarkers, targeted therapy

## Abstract

**Simple Summary:**

In ovarian cancer, about 70% of patients experience relapse despite primary cytoreductive surgery and platinum-based chemotherapy. The occurrence of resistance to chemotherapeutic agents remains a major obstacle in this context. Various factors are involved. The use of immunotherapy has widened the spectrum of therapeutic options in ovarian cancer. There are many different molecular parameters that have become relevant in recent years. The prognosis for relapsed patients is very poor, so an examination of such parameters and possible subsequent individualized therapy is desirable. In this work, we evaluate biomarkers that can or should be used in decision making about which therapy, but also which immunotherapy, is best. So far, there are some relevant biomarkers in ovarian cancer that can be used in primary therapy or recurrence therapy, such as genetic markers. The aim is to obtain additional parameters that can be used predictively, are of therapeutic relevance, and are prognostically relevant. The objective is to highlight the molecular mechanisms of the most promising targeted agents under clinical investigation to demonstrate their potential relevance in recurrent/refractory ovarian cancer.

**Abstract:**

Ovarian cancer (OC) has a high rate of mortality and is the fifth most common cause of death in females all over the world. The etiology is still unclear. Numerous factors such as smoking, obesity, and unhealthy diet may affect the risk of OC. Having a family history of breast and OC is one of the main risks for developing OC. Mutations of BRCA1/2 are associated with OC risk as well. The histopathological classification of OC reveals the four most common types: serous, clear cell, endometrioid, and mucinous; these are epithelial OC types, and other types are rare. Furthermore, OC can be subdivided into types I and II. Type I tumors are most probably caused by atypical proliferative tumors. Type II tumors include high-grade carcinoma of the serous type, carcinosarcoma, and carcinoma, which are not differentiated and generally originate from tubal intraepithelial carcinoma of the serous type. Typically, type I tumors are present in early stages, usually with good prognosis. Type II tumors are classified as high-grade tumors and are most often diagnosed at advanced FIGO stages with poor prognosis. High-grade serous OC accounts for 90% of serous OC. Tumor heterogeneity aggravates OC treatment. The standard care for primary epithelial ovarian cancer (EOC) is cytoreductive surgery followed by platinum-based chemotherapy. Neoadjuvant chemotherapy can be used in certain cases followed by cytoreductive surgery. The main prognostic factor is complete tumor resection. However, about 70% of patients relapse. Resistance to chemotherapeutic agents remains a major challenge in EOC treatment, in which many different factors are involved. In recent years, the examination of molecular parameters and their prognostic impact has become increasingly relevant in EOC, and furthermore, the use of immunotherapy has expanded the therapeutic range. As the clinical need is greatest for relapsed patients, this systematic review will focus on recent advances in molecular biology with prognostic and predictive markers and treatment options for recurrent/refractory OC. Inclusion criteria for the review: potential prospective or predictive biomarkers in preclinical or clinical use in relapsed and refractory OC, prognostic impact, clinical and preclinical trials, and immunotherapy. Exclusion criteria for the review: primary OC, no full text or abstract available, not the topic mentioned above, and text not available in English. Risk of bias: the included studies were evaluated descriptively for the topics mentioned above, and data were not compared with each other. The objective is to highlight the molecular mechanisms of the most promising targeted agents under clinical investigation to demonstrate their potential relevance in recurrent/refractory OC.

## 1. Introduction

In women, ovarian cancer (OC) is the fifth leading cause of cancer deaths [1]. In the United States, the American Cancer Society counted approximately 19,880 new cases of OC in 2022 [2], and approximately 12,810 women will die from it [2]. In primary epithelial ovarian cancer (EOC), the standard care is cytoreductive surgery combined with platinum-based chemotherapy [1]. Complete cytoreduction is the main prognostic factor, as the residual tumor mass has strongest impact on progression-free survival (PFS) and overall survival (OS) [3]. The etiology of OC is still unclear [4]. Numerous factors such as smoking, obesity, and unhealthy diet may affect the risk of OC, but they have a small role in OC [4]. Having a family history of breast and OC is one of the main risks for developing OC [4]. Mutations in BRCA1/2 are also associated with OC risk. The classification of OC is performed by the WHO [4]. Therefore, OC is classified into the following types: epithelial, mesenchymal tumors, mixed epithelial and mesenchymal tumors, sex cord-stromal tumors, germ cell tumors, monodermal teratoma, miscellaneous tumors, mesothelial tumors, soft tissue tumors, tumor-like lesions, lymphoid/myeloid tumors, and secondary tumors [4]. The most frequent type of OC is epithelial OC, found in approximately 90% of cases. The histopathological classification of OC reveals the four most common types: serous, clear cell, endometrioid, and mucinous; these are also epithelial OC types [4] with a varied frequency of subtypes (serous (52%), endometrioid (10%), mucinous (6%), and clear cell (6%) tumors) [5]. Furthermore, epithelial OC can be subdivided into types I and II [4,6] based on clinicopathologic factors with different clinical behavior. Type I tumors are most probably caused by atypical proliferative tumors [6]. Type II tumors consist of high-grade serous carcinoma, carcinosarcoma, and carcinoma, which are not differentiated and generally originate from tubal intraepithelial carcinoma of the serous type [6]. Typically, type I tumors are usually present in early stages and have good prognosis [6]. Type II tumors are classified as high-grade tumors and are most often diagnosed at advanced FIGO stages with poor prognosis [6]. High-grade serous OC accounts for 90% of serous OCs [6]. Tumor heterogeneity aggravates the OC treatment [7,8].

In past years, more effective therapeutic options using novel chemotherapeutic regimens and targeted agents have made significant contributions to improving the outcomes [1,9]. The standard care for relapsed OC is mostly systemic treatment. Recent evaluations have demonstrated that the serous OC subtype is more sensitive to chemotherapy than others. Clear cell and mucinous subtypes had significantly worse prognoses, which might reflect a resistance to the administered chemotherapeutic agents. However, effective and novel treatment strategies are needed to enhance prognosis in advanced EOC. In particular, the mechanisms of tumor progression and metastasis are only partially understood. The tumor microenvironment (TME) is important in OC progression [10]. The TME consists of many different components: the extracellular matrix, which consists of chemokines, inflammatory cytokines, integrins, matrix metalloproteinases, and other secreted molecules, and stromal cells, including cancer cells, cancer stem cells, pericytes, cancer-associated fibroblasts, endothelial cells, and immune cells [10]. These components are involved in tumor progression and improve tumor cell proliferation, invasion, and migration [10]. Some are involved in an increase in platinum resistance and increase relapse [10]. Macrophages, as components of the TME, can restructure the extracellular matrix and regulate components of the extracellular matrix by degrading the extracellular matrix, which might facilitate the tumor cell migration, invasion, and metastasis [10]. Exact connections and their impact are not yet known.

The analysis of OC at the molecular level leads to the finding of potential biomarkers for diagnosis and progression and for potential therapeutic targets in the field of angiogenesis and homologous recombination deficiency (HRD) [11,12]. There are already therapeutic approaches for this from trials. Even the discovery of cancer genes and mutations could potentially lead to advances in diagnosis and treatment [11,12]. The rationale is: the understanding of the tumor’s biology should be widened, which could make it feasible to discover prognostic biomarkers or predictors to facilitate the prognostic prediction of efficient therapy in refractory or resistant OC and that could help in treatment decisions. Patients with refractory or resistant OC have the greatest therapeutic need, and particularly for patients with refractory OC, the focus is on current advances in new therapeutic strategies for refractory OC currently under clinical evaluation that incorporate molecularly targeted agents to improve outcomes in advanced EOC. This review is restricted to recurrent/refractory EOC. The objective is to reveal the molecular mechanisms of the most promising targeted drugs currently under investigation in clinical research to demonstrate their potential relevance in recurrent/refractory OC.

## 2. Systematic Review

Platinum-based chemotherapies have high response rates of about 80%. Nevertheless, a relapse occurs in approximately 70% [9]. In selected cases, a cytoreductive surgery in relapsed platinum-sensitive OC should be considered [3,13,14]. This option is not suitable in non-platinum-sensitive/refractory recurrence. Other therapeutic options should be found to improve prognosis in recurrent disease.

With the use of immunotherapy in recent years, the spectrum of therapeutics was expanded with good prognostic impact in many cancers [15,16,17]. OC response to immunotherapy is limited [18]. The genome-wide sequencing in the past 10 years has gradually uncovered that of OC [19]. The identification of driver mutations in OC through projects such as The Cancer Genome Atlas (TCGA) made new therapeutic strategies available [19]. The predictive power of an immunotherapeutic response might be enhanced through assessing sensitive/resistant target treatment subpopulations stratified by biomarkers [18,20]. Further immunotherapeutic procedures like adoptive T cell therapy and vaccines are currently being investigated [7].

## 3. Cytotoxic Therapy

The situation of non-sensitive relapse is as follows: in platinum-resistant OC, most often a single-agent non-platinum cytotoxic therapy is used [3,14,21]. Response rates are about 13–50% [14]. If cancer becomes resistant or refractory to platinum drugs, less effective options are available [21]. Overall response rates (ORRs) and PFS intervals between these agents range from 10–15%, with a PFS of 3–4 months and OS around 12 months [21]. The overall response rates for second-line chemotherapy in recurrent OC are about 20–27%. Both primary and recurrence therapy have experienced improvements in recent years by the addition of so-called targeted therapies. Despite not being the gold standard of therapy (as mentioned above), targeted therapies are becoming increasingly important.

## 4. Angiogenesis

The use of VEGF inhibition (bevacizumab) combined with chemotherapy and as maintenance therapy leads to delaying in the disease progression in OC when administered alone or combined with chemotherapy in platinum-resistant OC [9]. VEGF overexpression is associated with disease progression and worse prognosis [9]. Nevertheless, many different mechanisms lead to tumor cells escaping therapeutic control, with the selection of tumor clones with increased expression of compensatory signaling pathways [1,9]. In platinum-resistant OC relapse with up to two prior chemotherapies, the application of bevacizumab added to chemotherapy leads to a significant improvement of PFS of 6.7 months compared to chemotherapy alone but does not change the OS [3,14,21]. There is approval for the application of bevacizumab added to second- or third-line non-platinum chemotherapy in platinum-resistant relapsed/refractory disease [14,22] and it has proven benefits for tumor remission and PFS. Therefore, various substances are currently being investigated to further improve outcomes [23]. Novel antiangiogenetic agents are under evaluation in combination therapies with drugs that target important pathways in tumor cells (DNA repair) or ICIs (PD-1/PD-L1) [9,24,25]. The application of tyrosine kinase inhibitor apatinib (VEGFR2 inhibition; phase I trial) achieved a short-term effect in recurrent platinum-resistant OC [9].

In recent years, other important angiogenesis drugs were evaluated. Angiopoietins 1 and 2 regulate angiogenesis and vascular remodeling by interacting with Tie2, a tyrosine kinase receptor, and inhibition of angiogenesis has shown promise in OC therapy [26]. This included trebananib as a non-VEGF-dependent angiogenesis pathway inhibitor. This therapeutic approach targets the angiopoietin–Tie2 complex pathway and inhibits the binding of angiopoietins 1 and 2 to Tie2. That is an alternative strategy overcoming VEGF-dependent antiangiogenesis-related resistance [26]. Currently, trebananib is being combined with ICI pembrolizumab (PD-1) in a phase Ib trial in advanced platinum-resistant OC [9]; results are still pending.

## 5. PARP Inhibitors

Further important therapeutic options are PARPis sensitizing tumor cells to DNA-damaging therapies. PARPis create double-strand breaks that cannot be precisely repaired in HRD tumors [27,28,29,30,31,32] and they are effective as maintenance therapy in recurrent platinum-sensitive OC, regardless of BRCA status [27]. However, PARPis are effective on the basis of a BRCA1/2 mutation in platinum-sensitive OC relapses [14] and more effective in platinum-sensitive relapse and negative BRCA1/2 status or platinum-non-sensitive relapse [3,14]. In primary treatment, PARPis should be administered after platinum-based chemotherapy [14] regardless of BRCA1/2 status.

Ten percent of OCs have a genetic disposition, so testing of mutations like BRCA1/2 is a standard of care. If there is no BRCA germline mutation, the potential for a somatic BRCA mutation should be examined [33]. A somatic or hereditary germline mutation in BRCA1/2 is detected in about 25–30% of high-grade serous OCs (HGSOCs) [14]. The TCGA has found that ∼50% of HGSOCs exhibit HRD, through many underlying mechanisms, some of which remain poorly defined [14,34]. The list of inherited mutations associated with OC means that up to one in four cases will have germline mutations, the majority of which result in HRD [35]. An additional 5–7% of OC cases will have somatic HRD [35]. Recently, some BRCA mutations and HRD have been shown as predictive biomarkers for PARPi treatment in OC [33] and as a clinical predictor for PARP sensitivity [14,36,37,38]. PARPis significantly extended PFS with underlying BRCA1/2 mutation in the germline and tumor (somatic) compared to BRCA1/2-negative HGSOC [14,37], so patients with HGSOC benefit from BRCA1/2 mutation and HRD positivity [14,31].

HRD is a phenotype that is characterized by the inability of a cell to effectively repair DNA double-strand breaks using the homologous recombination repair (HRR) pathway [32]. Loss-of-function genes involved in this process can sensitize tumors to PARPis and platinum-based chemotherapy, which target the destruction of cancer cells by working together with HRD through synthetic lethality [32]. The assessment of homologous recombination DNA repair (HRR) as a prognostic and predictive biomarker in HGSOC is leading to increasing discussion about the best means of defining and assessing HRD, both genotypic and phenotypic [34]. Currently, clinical-grade assays such as myChoice CDx and FoundationOne CDx are approved diagnostics which can find HRD-positive HGSOC in patients by finding a so-called “genomic scar” reflecting the underlying genomic instability. Moreover, tumoral HRD status can change over time [34]. The current challenge is that there is no standardized method to define, measure, and report HRD status using diagnostics in the clinical setting [32]. The definition of HRD varies widely [32]. HRD impairs normal DNA damage repair which results in loss or duplication of chromosomal regions, termed genomic loss of heterozygosity [35]. OC genomes can change a lot over time [39,40]. Furthermore, tumors could acquire mutations that affect their ability to perform DNA repair after testing [39,40]. This has to be considered [39,40]. Each patient should be tested for BRCA1/2 and PALB2 in primary OC [3], however, this does not predict prognosis or response to platinum-based therapy.

Various single-agent combinations for platinum-resistant OC are still being evaluated and some possible agents are listed in guidelines (National Comprehensive Cancer Network guidelines) [3]. In recent years, immunotherapy was increasingly used as well. Therapeutic options consist of immune checkpoint inhibitors (ICIs)/polyadenosine diphosphate-ribose polymerase inhibitors (PARPis)/targeted therapies [23,41,42,43,44]. Various substances that can be used or are promising are listed below (Table 1).

## 6. Immunotherapy and Potential Combinations

Immunotherapy is efficient in many relapsed cancers, but is moderately successful in OC. This therapy can be roughly divided into active, passive, or immunomodulatory [22]. Among others active immunotherapies are cancer vaccines, chimeric antigen receptor–T cell (CAR-T cell) therapy, or targeted therapies like trastuzumab [22,45,46]. “Passive immunotherapy” works by enhancing immune activity and indirectly targeting tumor cells. ICIs such as cytotoxic T-lymphocyte-associated protein 4 (CTLA-4) and programmed cell death protein 1 (PD-1)/PD-L1 monoclonal antibodies belong to passive immunotherapy [22,28,44,47,48,49,50,51,52,53,54,55]. Immunomodulators are substances regulating the activity of the immune system (i.e., ICIs) but also cytokines, agonists, and adjuvants [22,43,50,56]. Currently, the most commonly used immunotherapy drugs are ICIs [7,8,22,52,54,57,58,59], most commonly targeting PD-1/PD-L1 and CTLA-4, even though novel drugs (checkpoints) are being explored [15,16,25,28,30,41,42,47,48,49,53,56,57,58,59,60,61,62,63,64]. Various genes appear to have improved prognosis and may be more sensitive to immunotherapy, including PD-1 and CTLA-4 [12,62,64,65]. Other immune checkpoint molecules are under evaluation. In OC, expression of lymphocyte activation gene-3 (LAG-3) had significant prognostic impact on OS, but data are yet to be validated [16,66]. The effect of anti-LAG-3 antibodies alone or in combination with other immunotherapeutic agents should be investigated in clinical trials [67]. A phase II study of pembrolizumab demonstrates a response rate of around 10% for recurrent OC [3,7,22,42]. Dostarlimab (an ICI), that blocks the binding of PD-1 protein on T cells to the ligand PD-L1/2, has shown complete remission as the very first in colorectal cancer [23]. Trials in OC are ongoing (Table 1). “Active immunotherapy” achieves antitumor immune responses as a different treatment in OC [45]. Data are still lacking.

Below, current immunotherapeutic options targeting platinum-resistant OC in terms of opportunities and challenges in this research area [22] are presented. Many immunotherapeutic options are currently available: ICIs mono/dual therapy or ICIs +/− chemotherapy or ICIs + PARPi/antiangiogenesis.

## 7. Immune Checkpoint Inhibitors (ICIs)

There is still no valid predictor for immunotherapy use in OC [14,17,29,30,68]. Most OCs are infiltrated by activated T cells but show low remission rates with ICIs [69]. Despite the promising successes of immunotherapy, the application of single-agent ICIs in relapsed and/or refractory OC has been disappointing so far [17,61,68,70]. The Javelin phase Ib trial used anti-PD-L1 antibody (avelumab) in relapsed OC and resulted in a benefit for PD-L1+ compared to PD-L1-negative tumors. Consequently, PD-L1 could be used as a predictive biomarker and should be measured in this context [7,18,42,70]. Single-agent immunotherapy regardless of PD-L1 status in EOC has been disappointing, as demonstrated in the phase II KEYNOTE-100 trial (NCT02674061 https://clinicaltrials.gov/search?cond=NCT02674061 accessed on 29 October 2023). For pembrolizumab, a trend toward increased ORR was detected with higher PD-L1 expression [21,22]. Due to the promising results of ICIs, a combination of two ICIs was used. Below, therapy studies combining ICIs are presented: a phase I trial using ipilimumab/nivolumab [14] and a phase II trial using ipilimumab und nivolumab [3]. However, the addition of anti-PD-1 nivolumab and antilymphocyte-associated protein 4 (anti-CTLA-4) ipilimumab showed good results in platinum-resistant EOC, with an ORR of 34% [7,8], but no significant difference [22]. However, final results are expected [8]. An association between a response to nivolumab and PD-L1 expression was observed, suggesting that this biomarker has a limited role in assessing therapeutic response [8,47]. Efforts to optimize therapy effectiveness have included a combination of chemo- and immunotherapy or targeted drugs, including antiangiogenesis or a combination of various ICIs [70].

## 8. ICIs +/− Chemotherapy

A phase III study used ICI avelumab +/− chemotherapy vs. chemotherapy alone in platinum-resistant/refractory OC regardless of PD-L1 status (JAVELIN Ovarian 200 [39,71]. Neither combination significantly improved PFS or OS compared to PLD [22,71]. So, the anti-PD-1 application could be an option for chemotherapy in PD-1-positive tumors [72]. These results might improve patient stratification in further studies with ICIs in platinum-resistant/refractory OC. Further trials (AMBITION; KGOG 3045) enrolled patients with relapsed OC and subdivided the groups based on HRD and PD-L1 status. So, a biomarker-guided targeted therapy (multiarmed phase II trial) in platinum-resistant OC was performed [73] using PARPis, antiangiogenesis (VEGFRi), ICIs (PD-L1; anti-CTLA), and chemotherapy with respect to HRD status (positive/negative) [47,57,73]. All biomarker-driven targeted treatment combinations were manageable and without unexpected toxicities [73]. So, targeted therapies might be a new treatment option.

## 9. ICIs + PARP/Antiangiogenesis

One of the best strategies is co-targeting PARP and PD-1 as seen above. There are good reasons supporting this strategy, as HRD tumors have high PD-1 expression [37,70]. A further interesting option is the combination of ICIs and PARPis or antiangiogenesis. A phase I/II trial (KEYNOTE-162 combined pembrolizumab + niraparib in recurrent OCs and led to an ORR of 18% [22]. A further phase I/II trial (NCT02485990 https://clinicaltrials.gov/study/NCT02485990, accessed on 29 October 2023) used tremelimumab (a CTLA-4 inhibitor) vs. tremelimumab + olaparib [22]. This resulted in one patient with partial remission (PR) and nine patients with stable disease (SD) [22]. A phase III trial (NINJA) included nivolumab vs. cemcitabine or PLD [22]. No group had an improved OS. Based on the results, further improvements and new approaches are needed.

## 10. Promising New Approaches with Possible Future Relevance

New approaches are WEE-1 or ATR inhibitors or antibody–drug conjugates like mirvetuximab soravtansin consisting of a folate receptor alpha (FRα)-binding antibody. FRα is expressed in >80% of ovarian tumors and has limited distribution in normal cells [21]. A phase I trial resulted in a response rate of 39% with mirvetuximab soravtansin in platinum-resistant recurrent OC [14]. Promising results in phase II trials [21] did not lead to a significant PFS improvement using mirvetuximab soravtansin compared to chemotherapy in platinum-resistant EOC as demonstrated in a phase III trial [21,74]. Patient choice is critical to FRα-targeted agents; the high-expression subgroup did show improvement in the FORWARD I trial (NCT02631876) of mirvetuximab soravtansine, which included medium and high expression of FRα on targeted tumors, but lacked a PFS improvement vs. chemotherapy [21]. Meanwhile (November 2022), mirvetuximab soravtansine-gynx obtained Food and Drug Administration (FDA) approval for FRα-positive, platinum-resistant EOC with 1–3 prior systemic treatment options. At that point, further therapy options are restricted. One of the new strategies led to the use of a cell-cycle protein Wee1 (adavosertib) inhibitor in a phase II trial. Added to gemcitabine, it had better impact on tumor remission and OS compared to gemcitabine alone [39,75].

A further new approach is the use of PHI-101, a selective checkpoint kinase 2 inhibitor [76], which has shown antitumor activity in OC cell lines [76]. Besides that, an in vivo study suggests a synergistic effect of PHI-101 when combined with PARPis in OC. This phase Ia study could contribute to the development of a new combination regimen for OC therapy [76]. Results are still lacking.

Vaccination is another new approach. In platinum-resistant OC, intraperitoneal oncolytic viral immunotherapy (Olvi-Vec) was used in a phase Ib study. In a small cohort, an ORR of 9% was detected [22]. Currently, a phase III trial (NCT05281471) using Olvi-Vec in platinum-resistant OC is underway [22]. Another option is the use of dendritic cells (DCs) [77]. They have an impact on the TME as well, but there are no meaningful existing clinical studies of DC vaccine treatment in OC [77]. Little is known about the use of dendritic cell vaccinations in recurrent OC [77]. The murine monoclonal antibody oregovomab with high CA125 affinity [78] demonstrated in a study an increased number of patients with amplified CA125-specific CD8^+^T lymphocytes/mL which might have improved the therapeutic effect of standard of care + oregovomab over standard of care alone [78]. The prediction for oregovomab efficacy was a less suppressive immune environment [78]. Therefore, an increased CD8 content and decreased CD4/CD8 ratio are both favorable prognostic markers in IgG non-responders treated with personalized peptide vaccination [79].

Antitumor activity of CD3-T-cell-binding bispecific antibodies (TCBs) directed against the PAX8 lineage-driven HGSOC tumor antigen LYPD1 showed that anti-LYPD1 TCBs induce T cell activation and in vivo promote inhibition of tumor growth in LYPD1-expressing HGSOC. Taken together, these data demonstrate that bivalent TCBs targeting LYPD1 have compelling efficacy profiles that support their use in HGSOC treatment [80].

Since there has been no decisive success so far, further options are requested. Next to ICIs, one of the most tempting new options is targeting myeloid immune checkpoints like CD47 [7]. CD47 overexpression is seen in the majority of EOCs and is correlated with lower complete response rate to platinum-based adjuvant therapy [7]. The application of T-cell-recruiting therapies like ICIs or CAR-T cells (chimeric antigen receptors) suggests that the addition of anti-CD47 to T cell immunotherapies seems feasible [7]. Currently, a phase I trial is investigating the combination of Hu5F9-G4 (anti-CD47 antibody) with anti-PD-L1 avelumab (NCT03558139) [7]. CD24 might be a potential target as well, but data are outstanding [7]. The correlation of EOC cell metastasis with the levels of TGF-β1 or IL-10 suggested that immunosuppressive cytokines could be a novel target for inhibiting EOC progression [81].

## 11. Resistance to Therapeutic Agents

Resistance to chemotherapy drugs remains a major obstacle in OC treatment [1,7,42,52,53,82]. Numerous factors such as repair of DNA damage, inhibition of cell death, transition from epithelium to mesenchyme, and evasion of apoptosis have all been implicated in promoting chemoresistance [1,19,20,24,29,41,52,83]. Overcoming the resistance is challenging and approaches like sensitizing tumors to anti-PD-1 therapy are just beginning [41]. Homologous recombination repair (HRR) pathway deficiency (HRD) is implicated in HGSOC tumorigenesis and progression, as well as sensitivity to platinum chemotherapeutic agents. Many different factors are involved in their occurrence and the likelihood of platinum response is affected by histological subtype as well [84]. Low-grade serous, clear cell, or mucinous OCs have poor platinum response [84]. The histological subtype is a predictive biomarker as well and tumor heterogeneity is a huge challenge [7,8]. Evaluations detected a connection between molecular subtypes and prognosis but also their weakness in clinical implementation [85]. Platinum resistance is multifactorial, involving tumor genomics, intracellular mechanisms, and tumor microenvironment (TME) changes, making detection of such changes clinically challenging [22] and the underlying mechanisms are still unknown [7,20,42,84,86,87,88,89]. The TME of EOC is substantially altered by neoadjuvant chemotherapy as well [7]. The TME is an extremely valuable target for adjuvant interventions to improve immunotherapeutic effects [88,89]. There are different strategies targeting the TME [88,90]. There are few data on the TME in platinum-resistant OC [7]. Various genetic changes are associated with acquired resistance to platinum-based therapy, like inactivation of tumor suppressors RB1, NF1, RAD51B, and PTEN, reversions of BRCA1/BRCA2 mutations (germline or somatic), overexpression of MDR1, and CCNE1 amplification [84].

Innovative and efficient CAR targets are used in OC as well [46,91]. The TME constitutes an impediment to efficacy for OC-specific CARs [46,92]. So, a CAR-T cell strategy against CA125 and mesothelin (MSLN) could be an interesting approach in primary and recurrent OC [47,92]. Data are outstanding. Further specific antigens like NY-ESO-1, FR-alpha, MUC16, and p53 are tempting immunotherapeutic targets, based on their increased expression in OC, and are still under investigation [47].

To increase the effectiveness of immunotherapies in OC, it will be important to understand the reasons for local and systemic immune responses [87]. Many aspects of the TME are known, but more detailed investigations must be carried out in order to understand the processes involved in peritoneal carcinosis, primary tumour, and metastasis [87]. For example, genetic analysis demonstrated that many testicular cancer antigen subtypes are present in OC and a few are relevant in chemoresistance [19]. More details are not yet available. One study uncovered two immune subtypes that responded differently to immunotherapy and suggested that some HGSOCs may be vulnerable to immunotherapies or combination therapies [93]. It might be possible that PD-1/PD-L1 blockade combined with IL15 signaling can defeat resistance to cytokine therapy in OC [94]. Another evaluation uncovered immunomodulatory effects of niraparib that may sensitize tumors to ICIs [95]. Tumor antigen-specific and antigen-independent IgA responses counteract the growth of OC by directing coordinated tumor cell, T cell, and B cell responses [69]. It is possible that immunotherapies that enhance the B cell response are more effective than substances that enhance the T cell response. This may matter in tumors that are resistant to ICIs [69]. A study demonstrated that the exosome-related gene risk model can predict the prognosis of OC well and also guide the choice of immunotherapeutic approaches [96]. Ten immune-related genes were found that can be used as prognostic predictors of OC, thereby supporting clinical decisions and individualized therapies [97]. The meaning is still unclear. A study revealed that redundant immune checkpoint interactors were associated with positive results, providing new paths for the evaluation of molecular reasons behind effects of immunotherapy drugs, especially for immune checkpoints [98]. Further research is needed.

## 12. Importance of Biomarkers in Therapy Resistance

### Biomarker-Adapted Decisions/Trials

For better patient stratification, a biomarker-driven patient selection might improve therapeutic decisions. To improve the effectiveness of immunotherapies, reliable biomarkers should be detected [99]. Biomarkers such as PD-L1 expression and microsatellite instability (MSI) have a high validation level [8,49,51,60,61,68,99,100]. In HGSOC, PD-L1 expression is demonstrated in 90% of cases, with 30% having high expression [70], but the role of PD-L1 in predicting therapeutic response in HGSOC is still inconsistent [61,70,100]. For example, contrary to previous data, a substudy (IMagyn050 trial) found that homologous-recombination-deficient tumors did not show better PFS when adding atezolizumab (PD-L1 inhibitor) [100]. However, PD-L1 expression might be a potential prognostic biomarker for patient selection benefiting from anti-PD-1/PD-L1 immunotherapy. However, prospective studies are needed [101].

Microsatellite instability (MSI) is a predictive factor of immunotherapy response to ICIs [21,30,49]. Tumor genomes with deficient DNA mismatch repair (dMMR) demonstrate high microsatellite instability (MSI-H) [21,30,49]. However, the addition of pembrolizumab in OC with histologically/cytologically confirmed MSI-H/dMMR (from KEYNOTE-158 (NCT02628067 https://clinicaltrials.gov/search?cond=NCT02628067 accessed on 29 October 2023)) with DNA-mismatch-repair-deficient disease or MSI leads to an improved ORR of 33% [21]. In later therapy lines, PARPis were associated with improved ORR over traditional chemotherapies, but they require BRCA positivity or HRD [21,31]. Currently approved agents in EOC are rucaparib, niraparib, and olaparib [21]. In platinum-resistant OC, rucaparib demonstrated an ORR of 47% in a subgroup analysis of the ARIEL4 trial (NCT02855944 https://clinicaltrials.gov/search?cond=NCT02855944 accessed on 29 October 2023) [21]. Further, the QUADRA trial (NCT02354586 https://clinicaltrials.gov/search?cond=NCT02354586 accessed on 29 October 2023) found ORRs of 29% in platinum-resistant OC and 19% in platinum-refractory OC with niraparib [21]. Since trials for later therapy lines all excluded patients with previous PARPi application [21], the impact of exposure to PARPis in earlier lines has to be evaluated in further trials. Possibly, the neutralizing effect of PD-L1 might lead to a better effect of PARPis in platinum-resistant cancer [39]. A phase I/II trial in recurrent platinum-resistant OC received ICI pembrolizumab along with PARPi niraparib and resulted in a response of 19% without BRCA mutation compared to 5% with PARPis alone [39]. As well as PD-L1 and MSI, BRCA status is a predictive biomarker [8]: BRCA status is a well-known predictor of PARPi response and tumors with HRD show a higher tumor-infiltrating lymphocyte (TIL) infiltration [7,8]. TILs are important in reducing resistance to platinum agents [102]. An immunosuppressive microenvironment and poor outcome are seen in the detection of CD163^+^ tumor-associated macrophages (TAMs) or plasmacytoid dendritic cells [7,90]. The simultaneous infiltration of glucocorticoid-induced TNF receptor (GITR) with other immune bio- and T cell markers underlines an important role for anti-GITR drugs in combined immunotherapies [103]. Besides that, GITR expression on tumors may indicate the presence of a new cancer immunity evasion strategy what should be explored in future research [103].

The stratification of patients on the basis of marker genes specific for special phenotypes represents a promising procedure predicting prognosis or response to therapy [85]. To improve OS in recurrent disease, different options are discussed. The question is which combination of antiangiogenic agents and ICIs or PARPis + ICIs or the use of three drugs such as antiangiogenic drugs, PARPis, and ICIs is more effective. There is an ongoing trial in recurrent OC using a combination of bevacizumab, bevacizumab + atezolizumab, and bevacizumab + atezolizumab + aspirin (EORTC-1508/NCT02659384 https://clinicaltrials.gov/search?cond=NCT02659384 accessed on 29 October 2023) to explore the effectiveness of PARPis and ICIs [8] with outstanding data.

## 13. Biomarkers That Facilitate Treatment Decisions

It is important to improve prognosis by evaluating combination strategies in this field and, in the future, biomarkers should be available for patient selection [70]. Germline mutations in BRCA1/2 are the best-known risk factors for HGSOC. HRD is the most important factor in refractory OC. Today, there are options for maintenance therapy with PARPis together with HRD and HR-proficient tumors [38]. The induction of HRD by inhibiting PIK3CA to sensitize against PARPis is currently under evaluation [14]. Results of previous studies revealed that immunotherapy response could be better in various OC subtypes [70]. About 10% of clear cell tumors are MSI-H, and in contrast to serous OC they have higher PD-1 expression [36,70,104]. OC might respond better to immunotherapy since both the BRCA1/2 and TP53 mutation statuses correlate with increased PD-1/PD-L1 levels. In HGSOC, high PD-1/PD-L1 expression and TILs are correlated with good prognosis [68,70].

Several biomarkers of immunotherapy already tested in OC are listed below (Table 2): PD-L1 was studied as a biomarker predicting response to anti-PD-1 therapy in various tumors [60,70]. In OC, data on prognostic and predictive significance of PD-1/PD-L1 expression and the presence of TILs are inconsistent [31,48,60,68]. The detection of TILs in tumors is correlated with favorable prognosis in HGSOC [70,102], but it is not sufficient to predict immunotherapy response since different mechanisms can affect the function of TILs and reduce their response [18,36,63,70,102]. Tumor mutational burden (TMB) means the total number of somatic coding mutations in tumors [63,70,105]. Prospective data confirming TMB as a potential biomarker are still awaited. Higher TMB, neoantigen, and ITH may account for the poorer prognosis in platinum-based chemotherapy. Higher TMB was observed in platinum-resistant patients [105]. Whether platinum-resistant patients are better candidates for immunotherapy is still unclear [105].

The phase II trial KEYNOTE-158 (NCT02628067 https://clinicaltrials.gov/search?cond=NCT02628067 accessed on 29 October 2023) investigated the application of pembrolizumab in solid tumors with high TMB. The FDA has recently approved pembrolizumab in unresectable or metastatic solid tumors with high TMB [70]. Drugs targeting DNA repair processes and immunotherapy seem to be the most promising future strategy [70]. So far, none of the known biomarkers have been prospectively validated in controlled clinical trials [70], Table 2. Expression of targetable substances such as PD-L1 and indoleamine 2,3 dioxygenase (IDO) may help guide patient stratification. The co-expression of PD-L1 and IDO might be important for dual immunotherapy, but so far there are no clear data [106].

In OC, another study showed deficiency of tumor-expressed B7-H3 leads to an increase in antitumor efficacy of paclitaxel or PD-L1 blockade monotherapy rather than their combined chemoimmunotherapy [55]. Therefore, B7-H3 could be a potential predictive biomarker for patient stratification and a potential therapeutic agent in OC [55]. aeTSAs might be the best targets for HGSOC immunotherapy [107]. Findings about TILs and tumor cells might help in identification of therapy-refractory tumors [108] and might be helpful in personalized therapy as well [108].

## 14. Recent Advances in Molecular Biology for Refractory OC

The finding of molecular targets that can be treated with drugs is an important aspect for further effective cancer therapies. In recent years, research focused on finding targets that are essential for tumor cell viability or immune evasion [83]. So, tyrosine kinase receptors were found [83]. Meanwhile, there are many biomarkers found that can be targeted using existing therapeutics. Some of these biomarkers are: DNA methylation [84]; histone deacetylases [84]; DNA repair [84] like HRD; BRCA 1/2; mutation load [84]; immune cell subsets [84]. The clinical validation is still unclear and should be evaluated in further trials. Various genetic changes are associated with acquired resistance to platinum-based therapy as demonstrated above [84]. Furthermore, we discuss the potential of prognostic and predictive markers as anticancer therapeutic targets (Table 2).

However, assessing sensitive/resistant target populations of treatment based on stratification by biomarkers may improve the predictive power of response to immunotherapy. Such markers are tumor mutation burden, PD-L1, TIL, HRD, and neoantigen intratumoral heterogeneity [18]. In primary OC, there are many ongoing trials for prognostic and predictive biomarkers in ovarian cancers (NCT03010124), while data are pending [1]. So far, there are few prognostic biomarkers and they are still not sufficient. Known prognostic biomarkers are listed in Table 1.

There are a few predictive biomarkers such as BRCA and HRD for the use of PARPis [12,24,29,30,37,65,77], but further research is needed. High-HRD cancers present an immune-sensitive TME [36]. Homologous recombination repair (HRR) allows cells to fix damage in double-strand DNA [12,24]. Mismatch repair deficiency (dMMR) means cells cannot repair DNA mistakes from cell division. Regarding MSI [30,49] or high microsatellite instability (MSH-H), in the presence of dMMR microsatellites can become unstable. In 2017, the FDA approved the ICI pembrolizumab to treat cancers having dMMR or MSI-H, being the first time that tumor treatment was approved merely on the basis of tumor genetic characteristics. ICIs have inconsistent therapeutic impact on OC and further biomarkers of therapeutic utility are sought [100].

Regarding molecular mechanisms of the most promising targeted substances, a study revealed that immune cell infiltration is caused by chemokine receptor–ligand interactions within and across compartments [109]. Further research should evaluate various potential molecular aspects that shape the tumor immune phenotypes and potentially influence therapeutic ways to improve the benefit from immunotherapies [109].

NK cells may be a hopeful target in the future [110]. MSLN-CAR NK cells have robust specific antitumor activity, and mesothelin might be a potential target for CAR NK cells and could be used in OC treatment [91].

## 15. Recent Advances in Treatment Strategies for Recurrent/Refractory OC

Currently, studies of new combinations including immunotherapy seem to be the most appropriate in the future. Most running trials include anti-PD-1/PD-L1 antibodies [70]. The combination of immunotherapy and PARPis is still very important and numerous studies are ongoing. For instance, the MOONSTONE trial (NCT03955471), a phase II study to evaluate the addition of niraparib and dostarlimab (TSR-042) in platinum-resistant OC had sobering results but was interrupted. Therefore, the usage of immunotherapy with a tyrosine kinase inhibitor (TKI) seems to be associated with great results as presented in the phase II LEAP trial combining lenvatinib (a multikinase inhibitor) plus pembrolizumab [22,70] in previously treated participants with selected solid tumors (YY). The ORR was 32% in recurrent EOC [22,70].

Many phase I–III trials are currently ongoing in recurrent OC with different aspects and intentions, combining different therapies like ICIs, PARPis, antiangiogenesis drugs, and chemotherapy. Several current studies in platinum-resistant OC are shown in Table 1 and described in detail. The phase III MITO33 trial compares the efficacy of a PARPi (niraparib) plus PD-1 inihibitor (dostarlimab) vs. chemotherapy in platinum-resistant recurrent OC. Further phase III trials are combining ICIs and antiangiogenesis drugs. A phase III trial (NCT03353831) evaluates the use of atezolizumab + bevacizumab and chemotherapy vs. placebo plus bevacizumab and chemotherapy in recurrent OC. The KEYNOTE-B96 (NCT05116189) phase III trial uses an ICI (pembrolizumab) plus paclitaxel +/− antiangiogenesis (bevacizumab) for platinum-resistant recurrent OC. Furthermore, combinations of ICIs with targeted therapy like mitogen-activated protein kinase (MAPK) inhibitors or mitogen-activated protein kinase kinase (MEK) inhibitors are new strategies in clinical trials [22]. The use of another immunomodulatory drug is under evaluation. The Artistry-1 phase I/II trial (NCT02799095 https://clinicaltrials.gov/search?cond=NCT02799095 accessed on 29 October 2023) explored the usage of pembrolizumab + nemvaleukin alfa (ALKS4230) in heavily pretreated recurrent platinum-resistant OC. Positive results (ORR: 28.6%) led to a designation by the FDA [22]. Therefore, the phase III trial Artistry-7 (NCT05092360 https://clinicaltrials.gov/search?cond=NCT05092360 accessed on 29 October 2023) evaluates the use of nemvaleukin alfa (IL-2 receptor agonist) in combination with an ICI (pembrolizumab) in platinum-resistant OC. A phase III study (NCT04729608 https://clinicaltrials.gov/search?cond=NCT04729608 accessed on 29 October 2023) compares the use of AXL inhibitor batiraxcept (AVB-S6-500) in combination with paclitaxel vs. placebo + paclitaxel in platinum-resistant recurrent OC. The phase III trial EPIK-O (NCT04729387 https://clinicaltrials.gov/search?cond=NCT04729387 accessed on 29 October 2023) evaluates the efficacy of a PI3K inhibitor (alpelisib) + PARPi (olaparib) in platinum-resistant/refractory HGSOC, with no germline BRCA mutation detected. The MIRASOL phase III study stratifies patients by the level of FRα tumor expression and compares the efficacy of FRα-binding antibody (mirvetuximab soravtansine) vs. chemotherapy in platinum-resistant HGSOC (Table 1).

Further phase I–II trials are ongoing using biomarker-driven therapies, like the phase II trial BOUQUET/WO42178, using many different markers like PIK3CA/AKT1/PTEN/BRAF/NRAS/KRAS/NF1/ERBB2 to evaluate the efficacy and safety of VEGF/PARPis/ICIs/CDK4/6 inhibitors/endocrine receptor inhibitors/HER2/MEK inhibitors/AKT inhibitors in recurrent rare EOC (Table 1). The phase II Opal trial (NCT03574779) combines a triple treatment of novel substances, TSR-042 (anti-PD-1)/VEGF and PARPis, evaluating the efficacy in PARP-naive OC (Table 1) [22,70]. The phase II Bold trial combines a triple treatment of VEGF/PARP and PD-L1 inhibitors to assess the safety and efficacy in recurrent OC [22,70] (Table 1). A phase I trial with a drug blocking AXL activation in platinum-resistant cancers leads to remission when combined with paclitaxel [39,83]. Axl seems to be an important subject as a prognostic biomarker and target for antitumor therapy [83]. Axl overexpression is associated with bad prognosis and therapeutic resistance [83]. A further phase I/II trial (NCT04019288) combines an AXL inhibitor (AVB-S6-500) and an ICI (durvalumab) in platinum-resistant/recurrent OC (Table 1). Other trials evaluate triple therapies combining a VEGFi/PARPi and ICIs [39]. Several clinical trials are testing the efficacy of ICIs and PARPis in recurrent EOC (Table 1). Results are awaited. Since some mechanisms have not yet been clarified, moderate results can always be expected.

## 16. Limitations

There are a few limitations to this systematic review. In OC, there is so far only a small amount of literature on biomarkers and immunotherapy, which diminishes the known information. Most often, the demonstrated drugs or markers are approaches from preclinical or clinical studies with small numbers of cases that do not allow any conclusions or have broad prognostic significance. However, the biomarkers appear to be very relevant for certain subgroups.

## 17. Future Strategies

Molecular drug targets and biomarkers can be used to identify patients for specific therapies [111] and which subgroups benefit most from combined therapies of different targeted approaches. New combinations of immunotherapies for platinum-resistant OC are likely to be important in the future [22]. It is important to study the biology of platinum resistance to achieve an even more targeted and personalized therapy.

Some findings show that immunotherapies targeting LAG-3 may improve prognosis in OC, especially in combination with anti-PD-1/PD-L1 drugs [66]. Since LAG-3 and PD-L1 are usually expressed at low levels, the combination also has a weakened therapeutic effect [66]. High LAG-3 expression was seen in TILs in many solid tumors, including OC [67]. This might be important in the future for OC.

For better patient stratification, improved molecular and immunophenotypic biomarkers and better knowledge about the TME are needed to more accurately identify who will most benefit from immunotherapeutic approaches [49]. Exosomes are involved in various important cellular responses in the TME and contain a variety of genes related to OC immunotherapy that could be potential biomarkers for OC diagnosis and prognosis [112]. They have great therapeutic potential in OC, but there are still only preclinical studies [112]. The use of immunotherapy in recent years has led to an expansion of options and the results of ongoing and upcoming studies will explain the importance of genomic-based therapy in OC [104]. To what extent the options mentioned above of immuno-oncologic drugs in OC need to be considered should be the topic of future research. PD-1/PD-L1 inhibitors and strategies to improve benefit for these patients, including anti-PD-L1 adding other agents (cytotoxics, antiangiogenics, PARPis, targeted or other immunotherapies), as well as using these drugs earlier in disease or in biomarker-selected patients [68], could be part of the strategy. Further immune therapies like cell therapies (CAR-T cells, vaccines from dendritic cells) might be more important later in OC but there are currently solely early-phase studies [50]. New therapeutic strategies targeting cell-cycle checkpoints, including CHK1 inhibition (prexasertib), might improve clinical response and overcome therapeutic resistance [113]. Interestingly, prexasertib had a lasting single-agent activity in recurrent OC regardless of clinical characteristics, BRCA status, or prior therapies (including PARPis) [113]. Responders vs. non-responders had no apparent correlation with genomic changes, highlighting the need for further biomarker approaches to identify the responders [113]. Other immunomodulatory agents suppressing T-reg cells or immunosuppressive macrophages could augment their efficacy if PD-1 or CTLA-4 checkpoints are blocked. A phase II trial investigated the combination of two immunotherapies (CTLA/PD-1) added to PARPis in OC with at least one mutation in homologous repair genes [38]. These strategies might improve patient selection and stratification. Further research is needed identifying biomarkers in the ovarian TME, which is crucial for more effective, safe, and personalized DDSs [88].

CD27 agonism or antibody-mediated depletion of granulocytic cells leads to a better tumor control after vaccination with anti-PD-1 therapy, further improving effectivity [59]. These findings affirm the application of immunotherapies as a good option to identify possible combination approaches in OC [59]. Tumors can produce many different cytokines, immune mediators, classical neurotransmitters, hypothalamic and pituitary hormones, biogenic amines, melatonin, and glucocorticoids [114]. Cancers can escape the body’s regulatory mechanisms as well, but also gain the ability to affect local and systemic homeostasis [114]. Through this, tumors might take control of the central neuroendocrine and immune systems to reset the body’s homeostasis in a mode favoring its expansion at the expense of the host [114]. The importance of this knowledge is still unclear.

Further new immunotherapeutic strategies consist of tumor vaccines against tumor-specific antigens targeting tumor cells combined with ICIs [70]. Findings from ongoing trials will possibly transform the management of platinum-resistant OC soon [70]. There are a few upcoming trials combining VEGF plus chemotherapy like the AGO-ovar 2.42 phase III randomized trial of navicixizumab +/− paclitaxel (NCT05043402) compared to paclitaxel alone in platinum-resistant EOC. Another upcoming trial is combining PD-1 antibodies and PARPis after treatment with PARPis (AGO-ovar 2.35 pilot study, NCT05126342) to calculate the efficacy of the combination of dostarlimab and niraparib in EOC relapse. In recurrent platinum-resistant OC, there is a phase Ib trial (NCT03907527) with multigenic, autologous CAR-T cell therapy engineered to express a CAR specifically targeting MUC16. Enrollment is ongoing for this phase Ib trial, data are awaited. Preclinical trials on CAR-T generated chimeric antigen receptor (CAR) T cells targeting B7-H3 (B7-H3.CAR-Ts) [115]. This resulted in control of the growth of OC in vitro and in orthotopic and metastatic xenograft mouse models [115]. Also, it was found that 4-1BB co-stimulation encourages lower PD-1 expression in B7-H3.CAR-Ts and leads to improved antitumor activity when targeting tumor cells with expression of PD-L1 [115]. B7-H3.CAR-Ts significantly controlled tumor growth in a tumor model, but further research is needed in this topic [115]. Overcoming platinum or PARPi resistance is challenging as well [86].

Due to the high genomic complexity of HGSOC, molecular patient stratification is limited due to reduced predictive and prognostic validity [116]. Most promising trials seem to be combinations of chemotherapy, immunotherapy, antiangiogenics, PARPis, targeted therapy, and/or antibody–drug conjugates [116]. Further research approaches and precise knowledge of tumor biology would require samples from platinum-resistant OC [20] because there are differences between pre- and postchemo biopsies [7]. Due to the low response rate to ICIs in platinum-resistant OC, various combinations of anti-PD-1/PD-L1 with other ICIs targeting T cells (i.e., CTLA-4) or myeloid cells (CD47), antiangiogenic molecules, chemotherapy, PARPis, or radiation therapy are performed [7]. Recent clinical trials demonstrated the important impact of immunogenomic biopsy studies carried out longitudinally for evaluating predictive biomarkers and better research of cancer biology [7].

## 18. Methods

**Figure 1:**
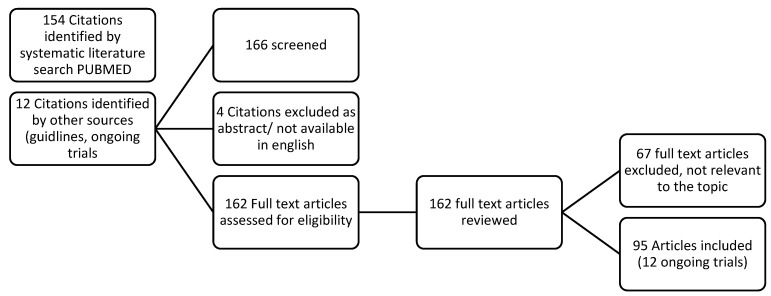
Literature search diagram. A total of 154 articles were extracted and a further 12 citations were identified from other sources. Therefore, 162 full texts were assessed and reviewed. A total of 67 full texts were excluded because they did not correspond to the intended topic or were not suitable for other reasons.

A systematic literature search was performed with PubMed and Cochrane Library for relevant references from 2018 through 11 May 2023 using ovarian cancer, refractory, biomarkers, and immunotherapy as search terms (Figure 1). The search string was ((((ovarian cancer[MeSH Terms]) OR (Ovarian cancer[Title/Abstract])) OR ((refractory ovarian cancer[MeSH Terms]) OR (refractory ovarian cancer[Title/Abstract]))) AND ((biomarkers[MeSH Terms]) OR (biomarkers[Title/Abstract]))) AND ((immunotherapies[MeSH Terms]) OR (immunotherapies[Title/Abstract])). Two reviewers collected data from each report and worked independently. Selection criteria leading to inclusion: all manuscripts found using the above search including the search terms ovarian cancer, refractory, recurrent, prognosis, biomarkers. Selection criteria leading to exclusion were no full text or abstract available, not relevant to the above-mentioned topic, text not available in English. Further articles like guidelines and ongoing and completed trials from clinicalgov.com were considered. The systematic review followed the recommendations of the Preferred Reporting Items for Systematic Reviews and Meta-Analyses (PRISMA). The protocol has not been registered. Risk of bias: the included studies were evaluated descriptively for the above-mentioned topics, data were not compared with each other.

The literature search diagram is included in Figure 1. A total of 154 articles were extracted and a further 12 citations were identified from other sources. Therefore, 162 full texts were assessed and reviewed. A total of 67 full texts were excluded because they did not correspond to the intended topic or were not suitable for other reasons.

## 19. Conclusions and Future Directions

We summarized the current standard of care and highlighted current aspects and studies in the field that may change the upcoming care of recurrent/refractory OC. So far, there are no biomarkers predicting prognosis and therapy effectiveness in OC, and the clinical impact is still not known. Based on the limited effect of single immunotherapies in platinum-resistant OC, ongoing trials evaluate combination therapies to improve therapeutic response and strategies overcoming therapeutic resistance. Results from preclinical/clinical trials suggest that at least a subgroup of OC patients benefits from a response to immunotherapy, either alone or combined with further drugs. Current prospective studies try to find potential biomarkers of response or resistance to immunotherapy. Therapeutic options are limited in platinum-resistant disease. At present, ICIs should be given in platinum-resistant disease in clinical trials. Further expansion of research on other biomarkers and alternative therapy options is necessary. Future research should focus on predictive and prognostic biomarkers, the reasons for platinum resistance, and OS in this special subgroup.

## Figures and Tables

**Table 1:** Current phases I–III studies are demonstrated using immunotherapy as therapeutic part in different combinations (dual/triple) in recurrent OC. The groups of active ingredients are added in brackets.

Current Studies of Immunotherapy	Phase of Studies	Drugs (Groups of Active Ingredients)
MITO 33 NCT04679064 https://clinicaltrials.gov/search?cond=NCT04679064 accessed on 29 October 2023	Phase III	Niraparib + dostarlimab vs. Pegylated liposomal doxorubicin/paclitaxel/gemcitabine/ topotecan +/− bevacizumab **PARP+ chemotherapy +/− VEGF**
AGO-Ovar 2.29 NCT03353831 https://clinicaltrials.gov/search?cond=NCT03353831 accessed on 29 October 2023	Phase III	Chemotherapy + bevacizumab +/− atezolizumab **Chemotherapy + VEGF +/− ICI**
AGO oVar 2.36 MIRASOL NCT04209855 https://clinicaltrials.gov/search?cond=NCT04209855 accessed on 29 October 2023	Phase III	Mirvetuximab soravtansine vs. paclitaxel/topotecan/pegylated liposomal doxorubicin **Antibody–drug conjugate targeting folate receptor α** (FRα) **vs. chemotherapy**
NCT04931342 (BOUQUET/WO42178)/(AGO 2.40) https://clinicaltrials.gov/search?cond=NCT04209855&intr=NCT04931342 accessed on 29 October 2023	Phase II	Ipatasertib/cobimetinib/trastuzumab emtansine/atezolizumab/bevacizumab/paclitaxel/giredestrant/abemaciclib/inavolisib/palbociclib/letrozole/olaparib/luteinizing hormone-releasing hormone (LHRH) agonists/cyclophosphamide **AKT inhibitor/VEGF/**ICI/PARP/chemotherapy
NCT05116189 (MK-3475-B96/KEYNOTE-B96/ENGOT-ov65) https://clinicaltrials.gov/search?cond=NCT04209855&intr=NCT05116189 accessed on 29 October 2023	Phase III	+/−Pembrolizumab + paclitaxel ± bevacizumab **ICI + chemotherapy +/− VEGF**
NCT05092360 Artistry-7 https://clinicaltrials.gov/search?cond=NCT04209855&intr=NCT05092360 accessed on 29 October 2023	Phase III	Nemvaleukin and pembrolizumab vs. pembrolizumab vs. nemvaleukin vs. pegylated liposomal doxorubicin (PLD) or paclitaxel or topotecan or gemcitabine **ICI/cytokine**
EPIK-O NCT04729387 https://clinicaltrials.gov/search?cond=NCT04729387 accessed on 29 October 2023	Phase III	Alpelisib + olaparib vs. paclitaxel or PLD **PI3K − Inhibitors + PARP**
LEAP-005 NCT03797326 (MK-7902-005/E7080-G000-224/LEAP-005) https://clinicaltrials.gov/search?cond=NCT03797326 accessed on 29 October 2023	Phase II	Lenvatinib +/− pembrolizumab **TKI +/− ICI**
BOLD trial (NCT04015739) https://clinicaltrials.gov/search?cond=NCT04015739 accessed on 29 October 2023	Phase II	Bevacizumab + olaparib + durvalumab (MEDI 4736) **VEGF + PARP + ICI**
OPAL trial (NCT03574779) https://clinicaltrials.gov/search?cond=NCT03574779 accessed on 29 October 2023	Phase II	TSR-042, bevacizumab, and niraparib **ICI/VEGF/PARP**
AVB-S6-500 NCT04729608 https://clinicaltrials.gov/search?cond=NCT04729608 accessed on 29 October 2023	Phase III	Paclitaxel +/− batiraxcept **Chemotherapy +/− Axl receptor tyrosine kinase inhibitors**
NCT04019288 AVB-S6-500 + durvalumab https://clinicaltrials.gov/search?cond=NCT04019288 accessed on 29 October 2023	Phase I/II	Batiraxcept + durvalumab **Axl receptor tyrosine kinase inhibitors + ICI**

**Table 2:** Many biomarkers that could be predictive or prognostic in OC patients. The table demonstrates the potential clinical role and therapeutic field of use.

	Clinical Role		Clinial Utility/Trial	
Biomarkers	Predictive/Prognostic Biomarker	Prognosis	Cancer Therapy	Clinial Utility
Folate receptor alpha (FRα) FOLR1	Predictive		Use of FRα-binding antibody mirvetuximab soravtansin	Chemoresistance; FDA approved for FRα-positive, platinum-resistant EOC
PD-1/PD-L1 expression Programmed cell death protein 1	Predictive Prognostic	PD-1/PD-L1 expression	Phase Ib trial used anti-PD-L1 antibody (avelumab) with improved prognosis in PD-L1-positive vs. PD-L1-negative tumors	High expression in HGSOC is associated with favorable prognosis
TILs	Prognostic	TILs		High expression in HGSOC is associated with favorable prognosis; reduces platinum resistance
BRCA	Predictive	BRCA1/2 positive	Use of PARPi	Predictive of response to PARPi
HRD	Predictive		Use of PARPi	HRD positivity is a clinical predictor for PARP sensitivity
MSI	Predictive			
PARP				Efficacy in recurrent platinum-sensitive OC regardless of BRC mutation
PTEN				
Tumor mutational burden (TMB)	Predictive		Pembrolizumab	Therapy with pembrolizumab in high TMB
TP53	Predictive			
AXL	Prognostic	AXL overexpression		Overexpression has worse prognosis; potential therapeutic target
VEGF	Prognostic	VEGF overexpression in tissue	Use of VEGF inhibitors like bevacizumab delaying disease progression	High expression of VEGF is associated with worse PFS
VEGFR2		VEGF overexpression in tissue	Tyrosine kinase inhibitor apatinib	Phase I trial; short-term effect
CTLA-4	Prognostic		T-lymphocyte-associated protein 4 antibody (ICI)	Combination of anti-PD-1 nivolumab and anti-CTLA-4 ipilimumab showed promising results in platinum-resistant EOC; combination with PARPi
WEE-1 inhibitors	Prognostic		Inhibitor of cell-cycle protein Wee1 (adavosertib) in a phase II trial	Improved OS compared to chemotherapy alone
ATR inhibitors				
Selective checkpoint kinase 2 (Chk2) inhibitor			PHI-101, an orally available, selective checkpoint kinase 2 (Chk2) inhibitor	Combined with PARPi in phase Ia trial
